# Inhibitors of the Transcription Factor STAT3 Decrease Growth and Induce Immune Response Genes in Models of Malignant Pleural Mesothelioma (MPM)

**DOI:** 10.3390/cancers13010007

**Published:** 2020-12-22

**Authors:** Moshe Lapidot, Abigail E. Case, Dalia Larios, Helen I. Gandler, Chengcheng Meng, Isidora Tošić, Ellen L. Weisberg, Michael J. Poitras, Prafulla C. Gokhale, Cloud P. Paweletz, Klaus Podar, Ravi Salgia, Srinivas V. Saladi, James D. Griffin, David A. Frank, Raphael Bueno, Martin Sattler

**Affiliations:** 1 Department of Surgery, Brigham and Women’s Hospital, Harvard Medical School, Boston, MA 02115, USA; mlapidot@bwh.harvard.edu (M.L.); DLARIOSCHAVEZ@mgh.harvard.edu (D.L.); rbueno@bwh.harvard.edu (R.B.); 2 Department of Medical Oncology, Dana-Farber Cancer Institute, Boston, MA 02215, USA; abigail.case@quinnipiac.edu (A.E.C.); helen.gandler@med.uvm.edu (H.I.G.); Chengcheng_Meng@DFCI.HARVARD.EDU (C.M.); Isidora_Tosic@dfci.harvard.edu (I.T.); Ellen_Weisberg@dfci.harvard.edu (E.L.W.); James_Griffin@dfci.harvard.edu (J.D.G.); David_Frank@dfci.harvard.edu (D.A.F.); 3 Department of Medicine, Harvard Medical School, Boston, MA 02115, USA; SrinivasVinod_Saladi@MEEI.HARVARD.EDU; 4 Experimental Therapeutics Core, Dana-Farber Cancer Institute, Boston, MA 02210, USA; MichaelJ_Poitras@DFCI.HARVARD.EDU (M.J.P.); Prafulla_Gokhale@DFCI.HARVARD.EDU (P.C.G.); 5 Belfer Center for Applied Cancer Science, Dana-Farber Cancer Institute, Boston, MA 02215, USA; CloudP_Paweletz@DFCI.HARVARD.EDU; 6 Department of Internal Medicine, Karl Landsteiner University of Health Sciences, 3500 Krems an der Donau, Austria; klaus.podar@krems.lknoe.at; 7 Department of Medical Oncology and Therapeutics Research, City of Hope, Duarte, CA 91010, USA; rsalgia@coh.org; 8 Massachusetts General Hospital Cancer Center, Boston, MA 02114, USA; 9 Massachusetts Eye and Ear Infirmary, Boston, MA 02114, USA

**Keywords:** malignant pleural mesothelioma, STAT3, targeted therapy, aberrant signal transduction

## Abstract

**Simple Summary:**

Malignant pleural mesothelioma (MPM) is characterized by the lack of effective long-term treatments and highly prevalent drug resistance. The paucity of potential therapeutic targets has led to dismal prognosis. We have examined the functional role of the signal transducer and activator of transcription 3 (STAT3) transcription factor in MPM. Even though highly specific STAT3 inhibitors have not yet come to fruition, we performed experiments targeting STAT3 expression and subsequently supported these experiments with small molecule drugs that were previously validated to target STAT3-dependent activation mechanisms. These drugs are United States Food and Drug Administration (FDA)-approved and showed efficacy in preclinical models of MPM at concentrations that can safely be achieved in humans. We also identified genes that strongly support the essential role of STAT3 in cell growth and are consistent with a role of STAT3 in immune suppression. Overall, the results establish a central role for STAT3 in tumor growth and encourage further expedient development of STAT3 pathway inhibitors for clinical use.

**Abstract:**

Malignant pleural mesothelioma (MPM) is an aggressive cancer defined by loss-of-function mutations with few therapeutic options. We examined the contribution of the transcription factor Signal transducer and activator of transcription 3 (STAT3) to cell growth and gene expression in preclinical models of MPM. STAT3 is activated in a variety of tumors and is thought to be required for the maintenance of cancer stem cells. Targeting STAT3 using specific small hairpin RNAs (shRNAs) or with the pharmacologic inhibitors atovaquone or pyrimethamine efficiently reduced cell growth in established cell lines and primary-derived lines while showing minimal effects in nontransformed LP9 mesothelial cells. Moreover, atovaquone significantly reduced viability and tumor growth in microfluidic cultures of primary MPM as well as in an in vivo xenotransplant model. Biological changes were linked to modulation of gene expression associated with STAT3 signaling, including cell cycle progression and altered p53 response. Reflecting the role of STAT3 in inducing localized immune suppression, using both atovaquone and pyrimethamine resulted in the modulation of immunoregulatory genes predicted to enhance an immune response, including upregulation of ICOSLG (Inducible T-Cell Costimulator Ligand or B7H2). Thus, our data strongly support a role for STAT3 inhibitors as anti-MPM therapeutics.

## 1. Introduction

Malignant pleural mesothelioma (MPM) is an aggressive cancer with a dismal prognosis. It is mainly associated with occupational exposure to asbestos and less frequently with prior radiation exposure. The incidence of MPM increases with age and in the United States has a median age of 62 years at diagnosis. Epithelioid histologic subtypes of MPM are the most common and least aggressive, followed by biphasic and sarcomatoid subtypes [1]. Biomarkers in epithelioid and biphasic MPM have been discovered that may help to predict outcome or disease recurrence after surgical resection [2]. Therapeutic options are limited, with median overall survival between 6 and 12 months after initial diagnosis, and thus novel targeted approaches are sought (see for review [3]).

Under physiologic conditions, the transcription factor STAT3 is activated transiently in response to cytokines and growth factors and regulates expression of genes controlling proliferation, survival, and self-renewal [4,5]. In many solid tumors and hematologic malignancies, signal transducer and activator of transcription 3 (STAT3) is constitutively activated, thereby driving the malignant behavior of tumor cells [6]. Activated tyrosine phosphorylated STAT3 was found in 61.4% (27/44) of archived MPM cases [7]. However, the exact role of STAT3 in MPM is not well-defined. Chemotherapy-resistant MPM cell lines show a functional and physical interaction between STAT3 and NFkB and stability of the STAT3-NFkB may be required for chemoresistance [8]. Additionally, low expression levels of Protein inhibitor of activated STAT3 (PIAS3), an endogenous inhibitor of STAT3 signaling, are associated with increased STAT3 activation and poor survival in MPM [9]. MPM itself is characterized by mutations in tumor suppressor genes, which are not directly related to STAT3 activation [10,11]. Thus, epigenetic regulation may play a larger role in the activation of STAT3 pathways. Our previous data suggest that STAT3 can also be infrequently activated in MPM by EPHA2 mutations with oncogenic characteristics [12].

The goal of this study was to define the role of STAT3 in MPM and to determine the efficacy of STAT3 pathway inhibitors, including atovaquone [13] and pyrimethamine [14,15], as potential treatments of MPM. We demonstrate that STAT3 is required for transformation of MPM cell lines and that pharmacological inhibition reduces malignant growth in vitro and in vivo. Our results further indicate that molecular mechanisms by which STAT3 inhibitors mediate anti-MPM activity include both direct effects via changing downstream target gene expression as well as indirect changes that may serve to enhance immune-targeted approaches.

## 2. Results

### 2.1. STAT3 Expression is Essential for Optimal Cell Growth in Malignant Pleural Mesothelioma (MPM) Cell Lines

**Figure 1:**
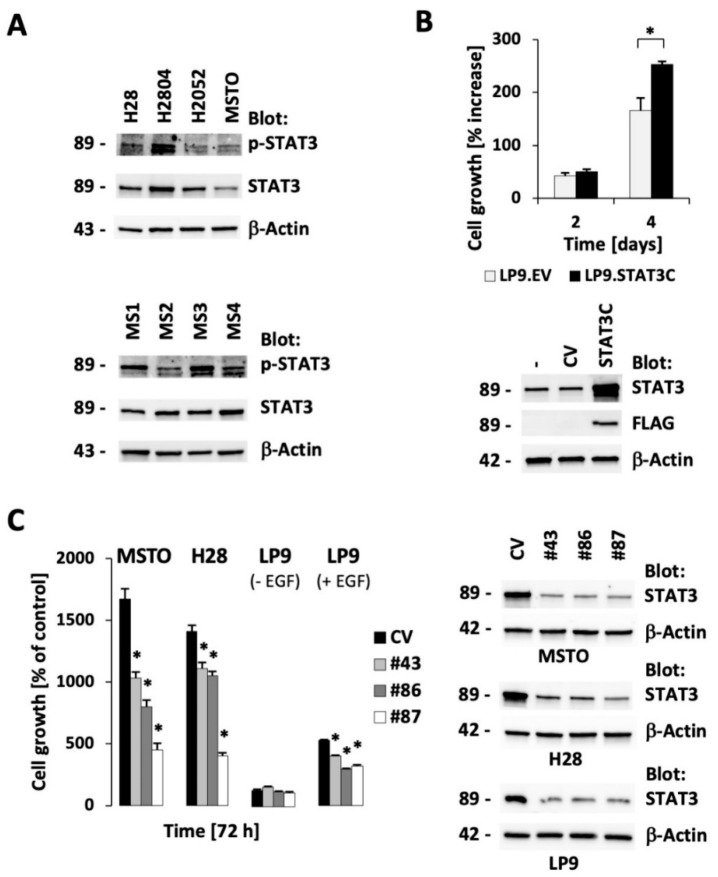
Signal transducer and activator of transcription 3 (STAT3) is phosphorylated in Malignant pleural mesothelioma (MPM) cells and its expression is required for optimal cell growth. (**A**) Protein expression of STAT3, phospho-STAT3 (Tyr705), and β-actin was measured by immunoblotting in established cell lines (H28, H2804, H2052, MSTO-211H (MSTO); top) and MPM primary-derived cell lines (MS1-MS4; bottom). (**B**) LP cells expressing active STAT3 (STAT3C) or containing a control vector (EV) were generated and growth was measured in a four-day culture (* significant differences with *p* < 0.05; top). Protein expression of STAT3, FLAG-tagged STAT3 and β-actin was determined by immunoblotting (bottom). (**C**) RNA silencing of STAT3 was performed in cell lines (MSTO-211H (MSTO), H28, LP9) and growth (*n* = 4) was measured using different small hairpin RNA (shRNA) expression constructs, as indicated (* significant differences with *p* < 0.005, left). Protein expression was measured by immunoblotting in response to STAT3 knockdown, as indicated (right). The molecular weights (in kDa) for each detected protein are indicted on the left of each immunoblot.

STAT3 expression and phosphorylation were evaluated in four established MPM cell lines (Figure 1A, top; see also Supplementary Materials Figure S1 for uncropped images). MSTO-211H (biphasic) and H28 (epithelioid) cells were used and compared to mesothelial LP9 cells, and, in some experiments, H2804 and H2052 were included where appropriate to support specific findings. STAT3 was readily detected in all of these models, and there was variable phosphorylation of STAT3 at Tyr705 as an indicator of activation, but not necessarily STAT3 dependency, with the highest in the H2804 cell line. Phosphorylated STAT3 was also readily detected in MPM cell lines newly derived from surgical MPM tumor specimens (Figure 1A; Table S1). New surgical MPM cell lines were tested for the expression of calretinin as a control for mesothelial-derived tissue [16,17]; additional controls included the expression of CD90 [18] or the absence of the hematopoietic marker CD45. Although calretinin was expressed in all of the cell lines, CD90 was absent in a patient with sarcomatoid disease phenotype (Figure S2), as was previously described [18].

It has previously been shown that growth of some MPM cell lines is dependent on STAT3 itself, which may reflect the requirement of cytokines such as IL-6, which signal through this transcription factor [19]. To determine whether activated STAT3 is sufficient to enhance the proliferation of nontransformed mesothelial cells, we expressed an activated form of STAT3, STAT3C, in the mesothelial LP9 cell line. Indeed, expression of activated STAT3 increased growth of these cells in the absence of epidermal growth factor (EGF) (52% increased growth versus control LP9.EV; *p* < 0.05), indicating a central role of this protein in the biology of these cells (Figure 1B). In order to further evaluate the necessity of STAT3 in MPM cells, we used three different hairpins targeting STAT3. We found this protein to be a critical element for cell growth in the MPM cell lines MSTO-211H and H28. In additional control experiments, we also show that the modest growth of LP9 cells is only affected by STAT3 knockdown in the presence of EGF (Figure 1C). Further, increased STAT3 phosphorylation depends on EGF stimulation in LP9 cells but its phosphorylation does not define a growth phenotype (Figure S3). Growth factor receptor mediated STAT3 activation can be mediated through JAK1 or JAK2 but inhibition of these kinases with ruxolitinib had minimal effect on growth of MPM or LP9 dells in the absence of added growth factor (Figure S4). In summary, consistent with previous observations, our data strongly support a role of STAT3 as a promising therapeutic target for MPM therapy.

### 2.2. The Small Molecule Drugs Atovaquone and Pyrimethamine Inhibit Growth of MPM Cells

**Figure 2:**
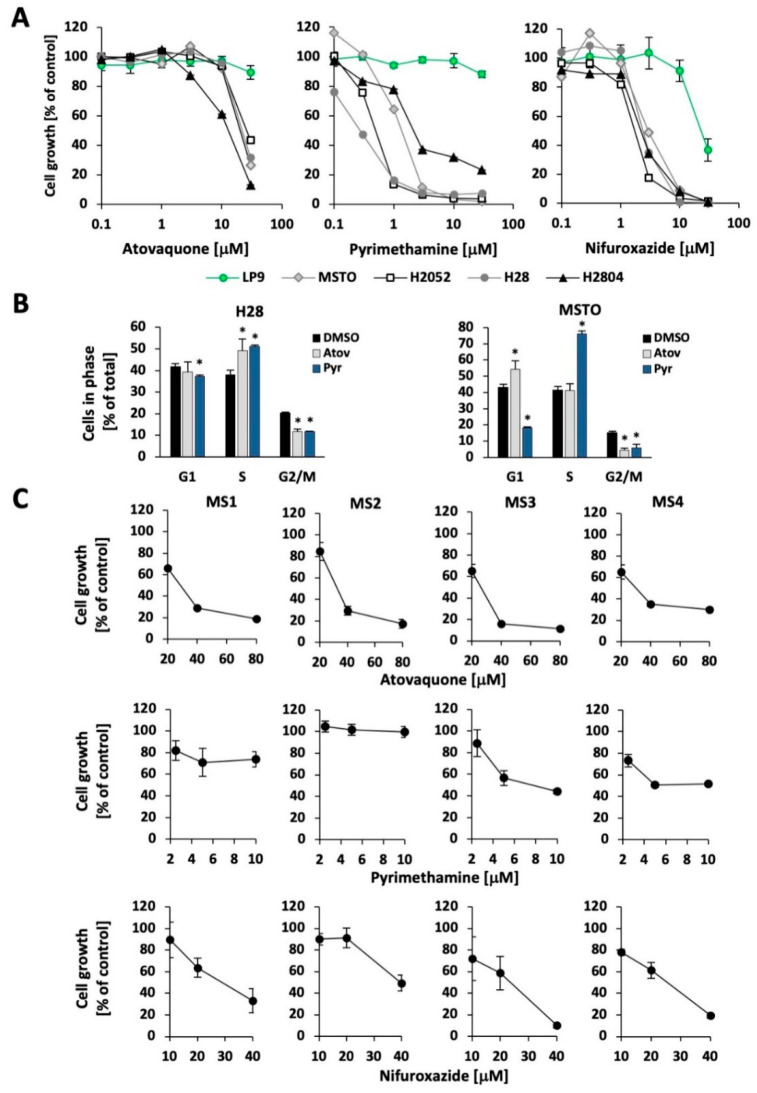
STAT3 pathway inhibitors reduce growth in MPM cell lines. (**A**) The MPM cell lines MSTO-211H (MSTO), H2052, H28, and H2804, as well as the nontransformed mesothelial cell line LP9, were treated with the indicated concentrations of atovaquone, pyrimethamine, or nifuroxazide for 72 h, after which viable cell number was quantitated (*n* = 4). (**B**) Cell cycle distribution was determined by propidium iodide staining in H28 and MSTO-211H (MSTO) cells in response to 30 μM atovaquone, 10 μM pyrimethamine or vehicle (control) after 18 h of treatment (*n* = 3) (* significant differences are indicated, *p* < 0.05). (**C**) MPM primary-derived cell lines (MS1-MS4) were treated with atovaquone, pyrimethamine, and nifuroxazide in a three-day assay (*n* = 4), as indicated.

The combination of cisplatin and pemetrexed represents the standard first-line therapy for MPM patients [20], with few alternative options. To determine the utility of STAT3 inhibitors in MPM, particularly those known to be safe in humans and readily translatable into clinical trials, we examined the effects of atovaquone [13], pyrimethamine [14,15], and nifuroxazide [21], all of which have been shown to decrease STAT3-dependent gene expression, albeit by different mechanisms. Each of the drugs led to a dose-dependent reduction in cell growth of the mesothelioma cell lines H28, H2052, H2804, and MSTO-211H (Figure 2A). By contrast, nontransformed LP9 mesothelial cells were found to be resistant to atovaquone or pyrimethamine (up to 30 μM) and had an IC_50_ for nifuroxazide that was approximately 10 times greater than for the MPM cell lines. The IC_50_ values for the STAT3 pathway inhibitor treatments have been summarized in Table S2. The concentrations at which the MPM cell lines showed a loss of viability are well within the range of concentrations of atovaquone [22] and pyrimethamine [23] achieved safely for prolonged periods in humans. Given their immediate availability for clinical translation, atovaquone and pyrimethamine were further investigated for their effects on the cell cycle distribution of MSTO-211H and H28 MPM cells. Whereas we consistently observed a significant decrease in the G2/M phase and accumulation in the S-phase, the effects of atovaquone on MSTO-211H cells were somewhat smaller and showed less accumulation in the S-phase, a decrease in the G2/M phase, and resulted in apparent G1 accumulation (Figure 2B). The results suggest that inhibition of multiple phases of the cell cycle may be an early effect of these drugs, with minor cell-specific differences. In contrast, increased cell death was not observed as an early event in these experiments. Cisplatin treatment is standard therapy in MPM and can be combined with pemetrexed. We looked to see whether cisplatin could also be combined with atovaquone or nifuroxazide and found that combinations of the drugs did not dramatically enhance or suppress either activity. In most cell line models, the combination of the highest drug concentrations used exceeded single agent activity, suggesting that combinations have the potential of increasing therapeutic efficacy in vivo (Figure S5). Pyrimethamine did not show a meaningful combination effect with cisplatin under these conditions (not shown).

The effect of these three STAT3 inhibitors was then assessed on the growth of primary-derived MPM cell lines. For simplicity, these experiments were performed at multiples of concentrations that induced at least 50% inhibition in cell lines (atovaquone: 20 μM; pyrimethamine: 2.5 μM; nifuroxazide: 20 μM). MS1-MS4 primary-derived cell lines were generally sensitive to atovaquone and nifuroxazide (IC_50_ < 40 μM), though they showed more variability in response to pyrimethamine (Figure 2C; see also Figure S6). For example, the primary-derived cell line MS2 displayed complete resistance towards pyrimethamine at every concentration tested, whereas growth was reduced by almost 50% in the cell line MS4.

### 2.3. Atovaquone Inhibits Growth in Preclinical Models of MPM

**Figure 3:**
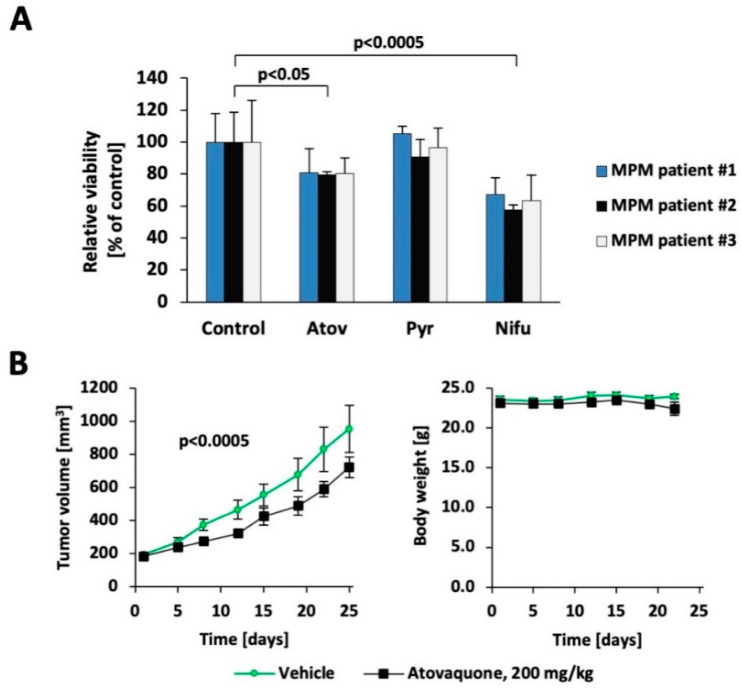
Targeting the STAT3 pathway in preclinical models of MPM. (**A**) Freshly isolated MPM patient spheroids of patient #1 and patient #3 were treated for 3d with 80 μM atovaquone, 6 μM pyrimethamine, 40 μM nifuroxazide or vehicle; patient specimen #2 received half the concentration. Changes in treatment groups were compared to their respective controls (*n* = 3). (**B**) Mice implanted with MS4 tumor fragments were treated with 200 mg/kg atovaquone or vehicle. Tumor volume and body weight were monitored.

3D cell culture models are thought to more closely mimic in vivo tumor physiology, including greater resistance to therapeutic agents. We found that atovaquone, pyrimethamine, and nifuroxazide all led to differential effects on MSTO-211H cells compared to nontransformed LP9 cells in 3D culture (Figure S7A,B), even though the same concentrations were toxic in monolayer culture settings (Figure 2A). We then tested the three drugs in three freshly isolated primary patient specimens and normalized the results to control-treated viable cells (Figure 3A). Under these experimental conditions, both atovaquone and nifuroxazide significantly reduced growth in primary cells, whereas pyrimethamine did not. Finally, the two United States Food and Drug Administration (FDA)-approved drugs atovaquone and pyrimethamine were tested in vivo, using xenografts in NOD/SCID IL2Rγ−/− (NSG) mice. In this model, tumor fragments from an earlier established model with low passage primary-derived MS4 cells were used and implanted in new mice. Tumor growth was followed for 25 days and showed a 38% reduction in tumor mass growth in the atovaquone-treated animals, using a once daily oral dosing regimen equivalent to standard human dosing (*p* < 0.0005) (Figure 3B, left). Reflecting the fact that atovaquone is extremely well-tolerated in humans, treatment itself did not alter the body weight of the mice (Figure 3B, right). Similar to atovaquone, pyrimethamine also significantly reduced tumor growth (*p* < 0.05), but, as expected from the in vitro data, the changes were smaller (Figure S7C).

### 2.4. Atovaquone and Pyrimethamine Target Genes in Cell Growth Pathways

**Figure 4:**
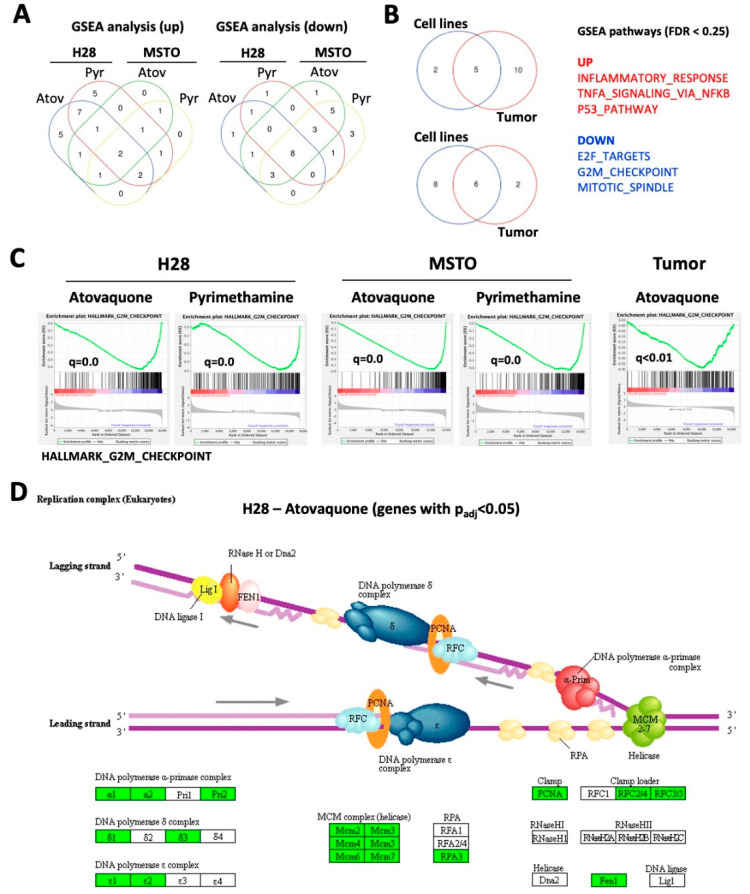
Atovaquone and pyrimethamine regulate pathways involved in cell growth. RNAseq data from H28 (*n* = 2), MSTO-211H (MSTO) (*n* = 2) or MS4 mouse tumors (*n* = 4) treated for 24 h with either pyrimethamine (10 mM), atovaquone (30 mM) or vehicle were used. (**A**) Gene Set Enrichment Analysis (GSEA) pathway analysis was performed to identify pathways that were upregulated or downregulated in treated cell lines. The Venn diagram indicates the number of common pathways in the various models (FDR < 0.25). (**B**) GSEA pathways that were identified in at least three cell line models were compared to GSEA pathways identified in atovaquone-treated MS4 tumors (FDR < 0.25). The Venn diagram identifies the number of common pathways and the three most prominent pathways are indicated. (**C**) Enrichment plots of the Hallmark G2M checkpoint pathway and q-values (FDR approach) for each model are shown. (**D**) Significantly changed genes (fold change >2 or <0.5; *p* < 0.05) in atovaquone-treated H28 cells were analyzed for their enrichment in the “replication complex (eukaryotes)” pathway using Pathview (upregulated genes—green, downregulated genes—red).

To identify the functional contribution of STAT3 to cell growth in MPM, we performed an RNAseq analysis of H28 and MSTO-211H cells in response to atovaquone and pyrimethamine. Targeting the STAT3 pathway with these drugs significantly altered the gene expression profile in both cell lines (*p* < 0.05; >2-fold change in expression) (Tables S3–S7). To specifically identify pathways enriched or depleted with treatment, we employed Gene Set Enrichment Analysis (GSEA) (false discovery rate (FDR) < 0.25). GSEA revealed that multiple pathways were commonly upregulated (n = 7) or downregulated (n = 14) in at least three of the four models (Figure 4A). To integrate these results with the effect of these drugs in vivo, we also performed RNAseq analyses with the tumors (n = 4) used in our in vivo mouse model using the primary-derived MS4 cell line. GSEA analysis from these results were then compared to the cell line data (Figure 4B). We identified a total of 17 pathways that were upregulated and 16 pathways that were downregulated in the mouse model. Three of the pathways most upregulated by the STAT3 inhibitors (inflammatory response, TNFα signaling, p53 pathway) likely reflect the role of STAT3 in the acute phase response and its cooperative effects with NFkB signaling and the DNA damage response. Three of the most prominently downregulated pathways (E2F targets, G2M checkpoint, mitotic spindle) reflect distinct transitions in the cell cycle, and may explain the divergent effects in cell cycle distribution seen with in vitro treatment of the MPM cell lines (Figure 2B and Figure 4C and Figure S8A, Table S8). Visualization of changes within this pathway was performed using the Pathview software within the “replication complex (eukaryotes)” and “cell cycle” models (Figure 4D and Figure S8B). This graph only analyzes genes that were found to be significantly changed (>2-fold change; padj < 0.05) in H28 cells treated with atovaquone, and which partially overlap with genes in the GSEA G2M checkpoint pathway. The results demonstrate that a majority of the genes defining cell cycle regulation are likely to be affected by treatment with these drugs.

### 2.5. Induction of Immune Response Genes by Atovaquone and Pyrimethamine in MPM Cells

**Figure 5:**
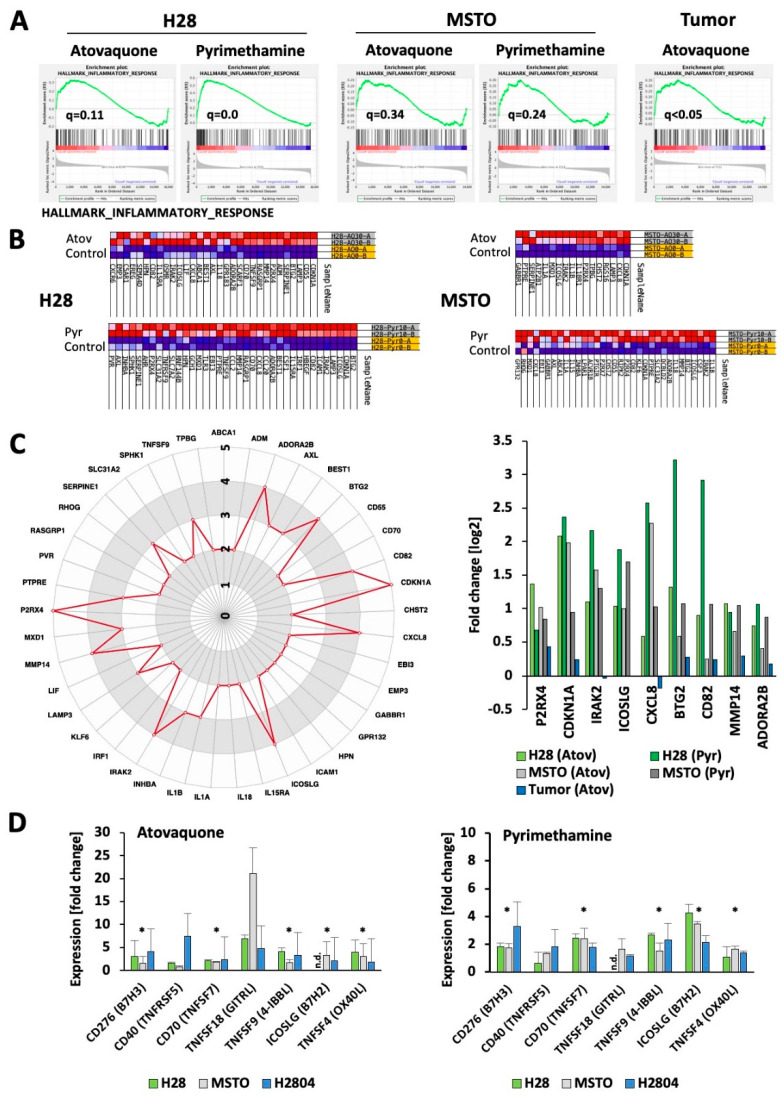
Atovaquone and pyrimethamine regulate immune pathway genes. (**A**) Enrichment plots of the Hallmark Inflammatory Response pathway and q-values (FDR approach) for different models treated with STAT3 pathway inhibitors, as indicated. (**B**) Enriched genes within the Hallmark Inflammatory Response pathways in H28 and MSTO-211H (MSTO) treated with either atovaquone (30 mM) or pyrimethamine (10 mM) are shown (upregulated genes—blue, downregulated genes—red). (**C**) Genes with the Hallmark Inflammatory Response pathway that are present in at least two of the five models are shown and scored according to their presence in each model (left). The relative changes in RNA expression in response to atovaquone (Atov) or pyrimethamine (Pyr) for genes that are present in at least four models are indicated (right). (**D**) The expression of additional immune pathway genes in response to 30 μM atovaquone or 10 pyrimethamine for 24 h in H28, MSTO-211H (MSTO) or H2804 cells was determined by RT-PCR (* significant differences with *p* < 0.05).

Disease-specific changes that depend on STAT3 signaling are difficult to predict. We found overlapping regulation of the “Inflammatory Response” pathway in our models (Figure 5A; see also the related TNFα signaling and p53 pathways (Figure S9)). The enrichment was apparent in H28 cells and our tumor model (q < 0.05), but somewhat weaker in MSTO-211H cells (q = 0.34 for atovaquone; q = 0.24 for pyrimethamine). A closer look at the genes that were enriched within this pathway demonstrated differences in the number and types of genes involved (Figure 5B). Out of the 88 unique genes, only 41 genes were found in at least two models. The spider chart identifies the most frequently changed genes (Figure 5C, left), including the cell cycle inhibitor gene *CDKN1A* and the purinergic receptor P2X 4 gene *P2RX4*, which were present in the enriched fraction of all models. Additional genes involved in at least four models include *IRAK2*, *ICOSLG*, *CXCL8*, *BTG2*, *CD82*, *MMP14* and *ADORA2B* (Figure 5C, right). Changes in these models are independent of significant changes within the gene expression dataset. Of special interest here are genes more closely related to the tumor immune response, such as *CXCL8*, which encodes for the chemokine IL-8 with a central role in regulating the innate immune response [24] as well as the *ICOSLG* gene which encodes for the Inducible T Cell Costimulator Ligand (CD275 or B7H2) [25]. The on-target effects of atovaquone and pyrimethamine were confirmed by measuring the expression of the STAT3 regulated genes MCL1 and BIRC5 [26,27] in treated H28, H2804, and MSTO-211H cells (Figure S10). Whereas both drugs led to a significant reduction in BIRC5 expression within this group of cell lines, only pyrimethamine significantly reduced MCL1 expression. However, atovaquone and pyrimethamine treatments were associated with a significant change (q < 0.25) in the HALLMARK_IL6_JAK_STAT3 gene expression signature in H28 cells.

It is known that drugs that suppress STAT3 signaling may decrease the effect of tumors in creating an immunosuppressive microenvironment, and may enhance both antigen presentation and effector T cell function [15,28]. In order to assess the functional role of atovaquone and pyrimethamine in eliciting an immune response in MPM cells, we compared the change in gene expression of a select number of B7 (*B7H2*, *B7H3*) and TNFR (*CD40*, *CD70*, *TNFSF4*, *TNSF9*, *TNFSF18*) family of immune genes in response to these drugs in H28, MSTO-211H, and H2804 cells (Figure 5D) using gene specific primers (Table S9). Both atovaquone and pyrimethamine significantly increased the expression of *CD276*, *CD70*, *TNFSF9*, *TNFSF4* and *ICOSLG*, though they had inconsistent effects on *CD40* and *TNSF18* expression. Overall, the results support the notion that targeting the STAT3 pathway through drugs such as atovaquone and pyrimethamine not only inhibits tumor growth but may also decrease the immunosuppressive microenvironment in MPM.

## 3. Discussion

There is a paucity of therapeutic targets in MPM and an urgent need for meaningful treatment advances, in particular for patients with the sarcomatoid phenotype. Given the evidence that the transcription factor STAT3 is often activated constitutively in this disease, we sought to evaluate STAT3 as a potential therapeutic target. Since inhibition of STAT3 is well-tolerated in normal tissue, this approach has the potential to have a high therapeutic index. To accelerate the potential for translational impact, we focused on STAT3 inhibitors that had already been approved for human use for other indications. STAT3 is an integral component in the transformation process of many malignancies, as it regulates genes involved in critical cellular processes, including proliferation, survival, self-renewal, invasion, and angiogenesis [6]. The mechanism of activation varies greatly, and it is not known how STAT3 is activated in MPM, but tyrosine kinases are rarely mutationally activated in this disease.

Due to the abundance of mutations in tumor suppressors, distinct mechanisms of activation are likely, including loss of expression of phosphatases and other negative regulators of this signaling pathway [29]. We show phosphorylation of STAT3 on its activation site in our experimental models. Further, an active form of STAT3 was sufficient to enhance cell growth in nontransformed LP9 mesothelial cells. The central role of STAT3 in the transformation of many cancers has elevated this transcription factor to a protein with oncogenic features [30]. In our models of MPM, STAT3 functions as a regulator of cell growth, which is supported by our pharmacological findings. The concentrations used in our in vitro models were well within the range that can be achieved in vivo for atovaquone and pyrimethamine [22,23]. Patient specimens grown in microfluidic 3D chambers allows malignant cells to grow in spheres, including cells present in the tumor microenvironment, such as stromal cells or hematopoietic cell types [31]. These primary cell results allowed us to establish efficacy in a range that is similar to what was achieved in cell line models. Even though higher concentrations were required for inhibition compared to a traditional monolayer model, at least in cell lines we were able to define a therapeutic window between MPM cells and our control cell lines. Furthermore, our in vivo models using a primary-derived cell line showed significant reduction in tumor growth. This model served as proof-of-concept and we did not attempt to optimize a potential dose effect. However, as mentioned above, higher concentrations are likely achievable in patients in vivo.

Differences in gene expression between our models may be attributed to the different drugs that were used but also to the genetic or phenotypical background of the experimental systems (MSTO-211H: biphasic; H28: epithelioid; MS4: biphasic). GSEA pathways that were found to be downregulated in our models (E2F targets, G2M checkpoint, mitotic spindle) affect different phases of the cell cycle. Thus, their inhibition would be expected to cause diverse effects on cell cycle distribution, as was seen experimentally (Figure 2B). In addition, STAT3 target genes regulate a variety of other cellular functions that can contribute to malignancy. This raises the possibility that STAT3 inhibitors in combination with conventional anticancer agents may be particularly useful in MPM. As noted, STAT3 inhibitors are likely to have a high therapeutic index when administered systemically. The fact that drugs to treat MPM can also be administered directly into the pleural space suggests that even greater therapeutic effects targeting STAT3 could be achieved.

Our gene expression data also showed increased enrichment within the p53 and NF-kB pathways. p53 is a known antagonistic regulator of STAT3, wherein loss of its function can lead to increased STAT3 activity [32]. Mutations in CDKN2A, encoding for p16INK4A and p14ARF, regulators of cell cycle and apoptosis through Rb and p53, are common in MPM and render cells resistant to apoptotic stimuli [11,33]. Increased p53 signaling would therefore be expected to be part of an important mechanism that targets STAT3-dependent growth in MPM. Additionally, similar to atovaquone and pyrimethamine in our models, the STAT3 inhibitor JSI-124 leads to activation of the NF-kB pathway in glioblastoma cells [34]. Overall, the gene expression data are consistent with STAT3 dependency in MPM.

Of special interest here is the additional immunoregulatory activity of STAT3, reflected by an upregulation of inflammatory response genes after STAT3 inhibition. Immunoediting and escape from attack by the immune system is one of the hallmarks of malignant transformation [35] and one of the current goals of immuno-oncology is to target escape mechanisms. Induction of inflammatory response genes has the potential to overcome these challenges and induce an antitumor immune response. Pyrimethamine, at least in breast cancer cells, is sufficient to induce an immune-stimulatory effect by inducing tumor infiltration of CD8+ T-cells with elevated Lamp1 [15]. Among other genes, we found upregulation of interleukin-8 (CXCL8) and Inducible T-Cell Costimulator Ligand or B7H2 (*ICOSLG*). Interleukin-8 is thought to promote tumor growth and may contribute to tumor growth in MPM in one murine model [36,37]. STAT3 inhibitors induced cell cycle arrest and reduced growth; it is not known how interleukin-8 influences the MPM tumor microenvironment in vivo, but interleukin-8 has the potential to retain antigen-presenting dendritic cells in the tumor [38]. The upregulation of *ICOSLG* is of particular interest as its product ICOS-L (ICOS-ligand) is a co-stimulatory B7 family member (B7H2), which binds to ICOS on T cells and may support anti-CTLA-4 immune checkpoint therapy [39]. Additional molecules tested for their increased expression in STAT3 inhibitor-treated cells are thought to support an antitumor response through regulating T-cell function.

Both atovaquone and pyrimethamine have previously been shown to be effective in murine xenotransplant models of STAT3-dependent cancers [13,14,15], and clinical trials using these drugs as STAT3 inhibitors, either alone or in combination with other agents, have commenced (ClinicalTrials.gov NCT03568994 and NCT01066663). Both drugs are orally bioavailable, with a long plasma half-life, and an excellent safety profile. These characteristics also allow these drugs to be easily combined with other anticancer agents, including immunomodulatory agents such as immune checkpoint inhibitors. MPM has generally been considered as an immunologically “cold” tumor. However, blocking STAT3 activity may decrease the immunosuppressive milieu and enhance immune effector function. Thus, combinations of STAT3 inhibitors such as pyrimethamine and atovaquone in conjunction with immune checkpoint inhibitors, may be particularly fruitful.

Overall, we define the STAT3 pathway as a regulator of cell growth with distinct immune effects and demonstrate the feasibility of targeting this mechanism in vivo. The STAT3 inhibitors used here have been repurposed for this task. They are known to be safe in humans and can easily be used in proof-of-concept clinical trials. Experiments to evaluate combination therapy with traditional therapy, with novel targeted approaches or with immune checkpoint inhibitors, are warranted.

## 4. Materials and Methods

Additional information can be found in the Supplementary Methods section.

### 4.1. Isolation of Primary MPM Cells

All patients in this study consented to tissue collection protocols, which were approved by the Institutional Review Board at Brigham and Women’s Hospital and the Dana-Farber Cancer Institute (Boston, MA, USA) (protocol number 98-063). MPM primary cells were isolated from discarded MPM tumors with apparent high tumor contents. Tumors were minced into pieces of approximately 2 mm^3^ and digested using the human Tumor Dissociation Kit (Miltenyi Biotec Inc, Auburn, CA, USA). Viable cells were filtered through a 70 μM nylon strainer (Falcon/Corning, Durham, NC, USA) and then maintained in RPMI 1640 containing 10% fetal bovine serum on plastic petri dishes (BD Falcon) for two passages before transfer to cell culture flask and further characterization.

### 4.2. MPM Spheroid Culture in Microfluidic Chambers

MPM tumor specimens, MSTO-211H or LP9 cells were used to prepare MPM spheroids analogous to previous preparations [40] In brief, fresh MPM tumor specimens were minced in Dulbecco’s Modified Eagle’s medium (DMEM) (containing 10% FBS, 100 mmol/L Na pyruvate, Corning CellGro, Tewksbury, MA, USA), 100 U/mL collagenase type IV (Life Technologies, Thermo Fisher Scientific, Waltham, MA, USA), and 15 mmol/L HEPES (Life Technologies, Thermo Fisher Scientific, Waltham, MA, USA). Visible red blood cells were removed using red blood cell lysis buffer (Boston Bio-Products, Ashland, MA, USA) and strained over 40 μm filters. Cells were maintained in ultralow-attachment tissue culture plates before injection into the culture chamber. Cell preparations or cell lines were pelleted and resuspended in type I rat tail collagen (2.5 mg/mL, Corning, Tewksbury, MA, USA) following the addition of 10× PBS with phenol red (pH 7.0–7.5). The spheroid–collagen mixture was then injected into the center gel region of the 3D microfluidic culture device. Collagen hydrogels were hydrated with media with or without indicated drugs or control treatments after 30 min at 37 °C. Microfluidic culture as well as live/dead staining and quantification of cells was performed as previously described [31,40].

### 4.3. Xenograft Mouse Models

In vivo mouse experiments were performed at the Dana-Farber Cancer Institute (Belfer Center for Applied Cancer Science) after approval by the Institutional Animal Care and Use Committee in an Association for Assessment and Accreditation of Laboratory Animal Care (AAALAC) accredited facility (protocol number 04-111). Female NOD/SCID IL2Rγ−/− (NSG) mice, 6 weeks old, were obtained from Jackson Laboratories (Bar Harbor, ME, USA) and injected subcutaneously with 10 × 10^6^ MS4 patient-derived cell lines with 50% Matrigel (Corning Life Sciences, Tewksbury, MA, USA). Tumors were allowed to grow to approximately 800 mm^3^, excised and ~3 mm^3^ tumor fragments were re-implanted in additional NSG mice for efficacy study. For efficacy study, MS4 tumors were allowed to establish to 200 ± 50 mm^3^ in size before randomization using Studylog software (Studylog Systems, San Francisco, CA, USA) into various treatment groups with 8–12 mice per group. Animals were treated with vehicle control, atovaquone (200 mg/kg) or pyrimethamine (75 mg/kg). Atovaquone (Mepron^®^) was diluted with water before use. Pyrimethamine was formulated in 10% NMP (N-Methyl-2-Pyrrolidone) and 90% PEG300. Animals were treated by once daily oral gavage for 22 days. Tumor volumes were determined from caliper measurements by using the following formula: tumor volume = (length × width^2^)/2. Tumor volumes and body weights were measured twice weekly. A two-way ANOVA followed by Tukey’s multiple comparison test was used to determine the significance of differences in the growth of tumor xenograft following treatments. Statistical comparisons were performed using GraphPad Prism v8.4.3 (Prism, San Diego, CA, USA). A *p*-value of less than 0.05 was considered significant.

## 5. Conclusions

In this study, we used gene expression profiling as an exploratory tool to define the STAT3 pathway for the first time as a regulator of cell growth with distinct immune effects in models of MPM. We also demonstrate the feasibility of targeting this mechanism in vivo. The STAT3 inhibitors used here, which are known to be safe in humans and can easily be used in proof-of-concept clinical trials, have been repurposed for this task. This is of interest since MPM is thought to be a “cold” cancer with relatively little infiltration of immune cells. Our results suggest that STAT3 is likely to induce localized immune suppression, and both STAT3 inhibitors, atovaquone and pyrimethamine, resulted in the modulation of genes predicted to enhance an immune response. Importantly, the identified immune targets have high potential relevance for future immunotherapy and have not been described before. Studies to evaluate combination therapy of STAT3 inhibitors with traditional therapy, with novel targeted approaches or with immune checkpoint inhibitors are warranted.

## Supplementary Materials

The following are available online at https://www.mdpi.com/2072-6694/13/1/7/s1: Supplementary Methods, Figure S1: Uncropped Western Blot images; Figure S2: Characterization of MPM primary-derived cell lines; Figure S3: Cell growth and STAT3 phosphorylation in LP9 cells in response to EGF; Figure S4: Cell growth of MPM cell lines in response to ruxolitinib; Figure S5: Combination of cisplatin with atovaquone or pyrimethamine in MPM cell lines; Figure S6: Cell growth of MPM primary-derived cells in response to atovaquone, pyrimethamine, nifuroxazide, and napabucasin; Figure S7: Targeting the STAT3 pathway in preclinical models of MPM; Figure S8: GSEA pathways downregulated by atovaquone and pyrimethamine; Figure S9: GSEA pathways upregulated by atovaquone and pyrimethamine; Figure S10: Atovaquone and pyrimethamine regulate the expression of STAT3 target genes; Table S1: Patient information; Table S2: IC_50_ values for STAT3pathway inhibitors; Table S3: Tumor (MS4)-atovaquone; Table S4: H28-pyrimethamine; Table S5: H28-atovaquone; Table S6: MSTO-pyrimethamine; Table S7: MSTO-atovaquone; Table S8: Common up- and downregulated GSEA pathways; Table S9: Primers.

## Abbreviations

MPM	Malignant Pleural Mesothelioma
STAT3	Signal Transducer and Activator of Transcription 3
ICOSLG	Inducible T-Cell Costimulator Ligand
NF-κB	Nuclear Factor Kappa-Light-Chain-Enhancer of Activated B Cells
EPHA2	Ephrin Type-A Receptor 2
NOD/SCID	Nonobese Diabetic/Severe Combined Immunodeficiency
GSEA	Gene Set Enrichment Analysis
EV/CV	Empty Vector/Control Vector
