# Supplementary material for: Inhibitors of the Transcription Factor STAT3 Decrease Growth and Induce Immune Response Genes in Models of Malignant Pleural Mesothelioma (MPM)
Source: Cancers (Basel). 2020 Dec 22;13(1):7. doi: 10.3390/cancers13010007 (PMC7792575; doi:10.3390/cancers13010007)
Supplement: Supplementary file 1 [file cancers-13-00007-s001.zip › cancers-100962-SI.pdf]

Supplementary Methods

Cell culture

Human MPM cell lines MSTO-211H (MSTO), H28, H2804 and H2052 were grown in RPMI 1640 (GIBCO) containing 10% fetal bovine serum (FBS; GIBCO). Cell lines were from ATCC and were either used within 6 months of culturing or submitted for cell line authentication within 6 months of use through cell line short tandem repeat (STR) profiling (Molecular Diagnostics Laboratory, Dana-Farber Cancer Institute). Human HEK-293T/17 cells were used for production of lentiviruses and mesothelial LP9.TERT cells [1] (kindly provided by Dr. M.R. Ramsey, BWH) were compared to MPM cell lines. LP9.TERT cells were also transfected with a control vector (EV) or vector containing a truncated constitutively active from of STAT3 (STAT3C [2]). In some experiments, cells were maintained in RPMI 1640 supplemented with 10% FBS and treated with STAT3 pathway inhibitors, including atovaquone (Selleckchem), pyrimethamine (Selleckchem), nifuroxazide (Selleckchem), napabucacin (Selleckchem) or the JAK1/2 inhibitor ruxolitinib (Selleckchem).

Cell growth

Cell concentrations were determined by trypan blue (Sigma-Aldrich) exclusion and cell growth was measured over time with the CellTiter-Glo Luminescent Cell Viability Assay Kit (Promega).

Targeted knockdown

Targeted knockdown was performed as described previously [3], using lentiviral constructs. Three constructs, each containing shRNA against STAT3 (TRCN0000020843 (#43), TRCN0000329886 (#86) and TRCN0000329887 (#87); Sigma-Aldrich), were used and a lentiviral construct containing shRNA against GFP [4] (Addgene). Lentiviruses were generated by co-transfecting HEK293T cells with viral packaging vectors pMD2.G and pCMVΔ8.91, as well as shRNAs using the TransIT-293 transfection reagent (Mirus). Cells were infected in the presence of polybrene (16 μg/mL; EMD Millipore) and 24h later selected for three days in medium containing puromycin (1 μg/mL; Sigma-Aldrich).

Immunoblotting

Immunoblotting was performed as described previously using a standard chemiluminescence technique [5] (for original images see **Supplementary Figures S10).** Rabbit polyclonal antibodies against phospho-STAT3 (Tyr705) (#9131; Cell Signaling), mouse monoclonal antibodies against β-actin (12H8; Sigma-Aldrich), STAT3 (124H6; Cell Signaling), FLAG (M2; Sigma-Aldrich), and rabbit monoclonal antibody against calretinin (SP13; ThermoFisher) were used to measure protein expression.

Cell cycle analysis

Cells were fixed with 70% (v/v) ethanol and incubated on ice for 30 minutes. Fixed samples were stained with propidium iodide solution (50 μg/mL propidium iodide, 1% NP40, 10% sodium citrate; Sigma-Aldrich) at 4°C for at least 15 minutes. Cell cycle parameters were determined by flow cytometry. The DNA analysis program ModFit LT (Verity Software House) was used to determine cell cycle distribution.

Analysis of CD90 and CD45 expression

Expression of CD90 (FITC labeled clone 5E10, BD Biosciences) and CD45 (PE labeled clone HI30, BD Biosciences) on MPM cell lines was determined by flow cytometry using standard methods.

RNA sequencing (RNAseq) analysis

Total RNA was isolated from cells using TRIzol (Ambion by Life Technologies) followed by RNeasy purification (RNeasy kit, Qiagen). Deep sequencing of RNA (RNAseq) and gene expression analyses were performed at Genewiz (South Plainfield, NJ). Gene Set Enrichment Analysis (GSEA) [6,7] was performed against signature databases (c2.cp.kegg.v6.2.symbols.gmt and h.all.v6.2. symbols.gmt). Pathview analysis [8,9] was done using the online version of the program (https://pathview.uncc.edu/).

Quantitative RT-PCR

RNA was harvested using TRIzol (Ambion by Life Technologies) followed by RNeasy purification (RNeasy kit, Qiagen). Complementary DNA (cDNA) was generated using the TaqMan Reverse Transcription Reagents (Applied Biosystems), and qRT-PCR (quantitative reverse transcription polymerase chain reaction) was performed using primers against CD276, CD40, CD70, TNFSF18, TNFSF9, ICOSL, TNFSF4 (**Supplementary Table S9)**. Data are expressed as mean fold change ± standard deviation of at least two independent experiments.

Statistical analysis. For statistical comparison between test and control groups, the Student's *t*-test was used, unless related groups were analyzed, then the paired-sample *t*-test was applied. Changes were calculated as the percent change relative to the control average. Error bars represent standard deviation (SD) of at least four independent experiments. Error bars represent standard deviation of at least four independent experiments, unless otherwise indicated. Each key experiment was replicated at least once or confirmed by related methodologies, if feasible. Shown are representative experiments.

References

1. Dickson, M.A.; Hahn, W.C.; Ino, Y.; Ronfard, V.; Wu, J.Y.; Weinberg, R.A.; Louis, D.N.; Li, F.P.; Rheinwald, J.G. Human keratinocytes that express hTERT and also bypass a p16(INK4a)-enforced mechanism that limits life span become immortal yet retain normal growth and differentiation characteristics. *Mol Cell Biol* **2000**, *20*, 1436-1447, doi:10.1128/mcb.20.4.1436-1447.2000.
2. Bromberg, J.F.; Wrzeszczynska, M.H.; Devgan, G.; Zhao, Y.; Pestell, R.G.; Albanese, C.; Darnell, J.E., Jr. Stat3 as an oncogene. *Cell* **1999**, *98*, 295-303, doi:10.1016/s0092-8674(00)81959-5.
3. Bhanot, H.; Weisberg, E.L.; Reddy, M.M.; Nonami, A.; Neuberg, D.; Stone, R.M.; Podar, K.; Salgia, R.; Griffin, J.D.; Sattler, M. Acute myeloid leukemia cells require 6-phosphogluconate dehydrogenase for cell growth and NADPH-dependent metabolic reprogramming. *Oncotarget* **2017**, *8*, 67639-67650, doi:10.18632/oncotarget.18797.
4. Sancak, Y.; Peterson, T.R.; Shaul, Y.D.; Lindquist, R.A.; Thoreen, C.C.; Bar-Peled, L.; Sabatini, D.M. The Rag GTPases bind raptor and mediate amino acid signaling to mTORC1. *Science* **2008**, *320*, 1496-1501, doi:10.1126/science.1157535.
5. Walz, C.; Crowley, B.J.; Hudon, H.E.; Gramlich, J.L.; Neuberg, D.S.; Podar, K.; Griffin, J.D.; Sattler, M. Activated Jak2 with the V617F point mutation promotes G1/S phase transition. *J Biol Chem* **2006**, *281*, 18177-18183, doi:10.1074/jbc.M600064200.
6. Mootha, V.K.; Lindgren, C.M.; Eriksson, K.F.; Subramanian, A.; Sihag, S.; Lehar, J.; Puigserver, P.; Carlsson, E.; Ridderstrale, M.; Laurila, E., et al. PGC-1alpha-responsive genes involved in

oxidative phosphorylation are coordinately downregulated in human diabetes. *Nat Genet* **2003**, *34*, 267-273, doi:10.1038/ng1180.
7. Subramanian, A.; Tamayo, P.; Mootha, V.K.; Mukherjee, S.; Ebert, B.L.; Gillette, M.A.; Paulovich, A.; Pomeroy, S.L.; Golub, T.R.; Lander, E.S., et al. Gene set enrichment analysis: a knowledge-based approach for interpreting genome-wide expression profiles. *Proc Natl Acad Sci U S A* **2005**, *102*, 15545-15550, doi:10.1073/pnas.0506580102.
8. Luo, W.; Brouwer, C. Pathview: an R/Bioconductor package for pathway-based data integration and visualization. *Bioinformatics* **2013**, *29*, 1830-1831, doi:10.1093/bioinformatics/btt285.
9. Luo, W.; Pant, G.; Bhavnasi, Y.K.; Blanchard, S.G., Jr.; Brouwer, C. Pathview Web: user friendly pathway visualization and data integration. *Nucleic Acids Res* **2017**, *45*, W501-W508, doi:10.1093/nar/gkx372.

Supplementary Figures

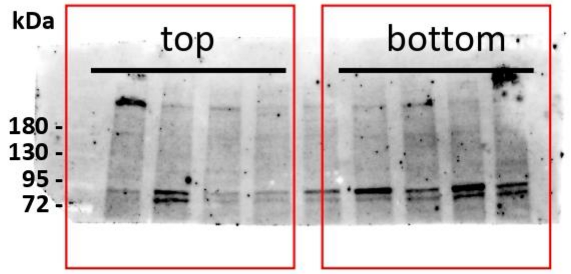

Figure 1A: Blot: p-STAT3

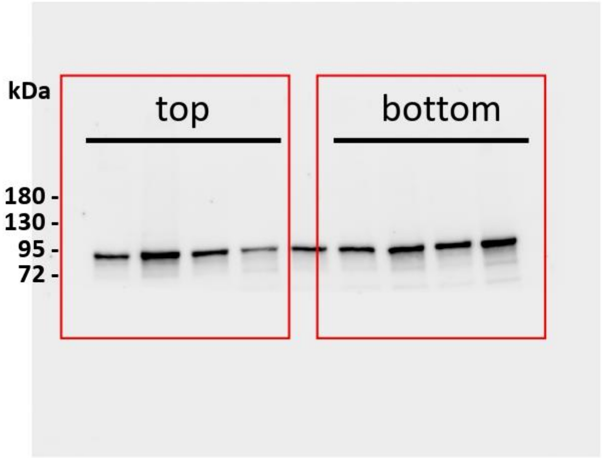

Figure 1A: Blot: STAT3

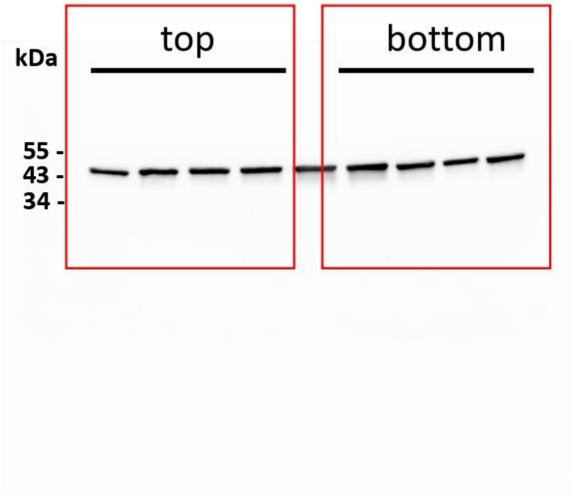

Figure 1A: Blot: β-Actin

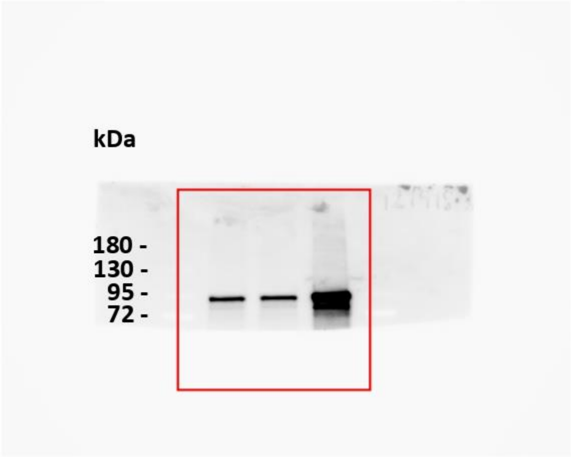

Figure 1B: Blot: STAT3

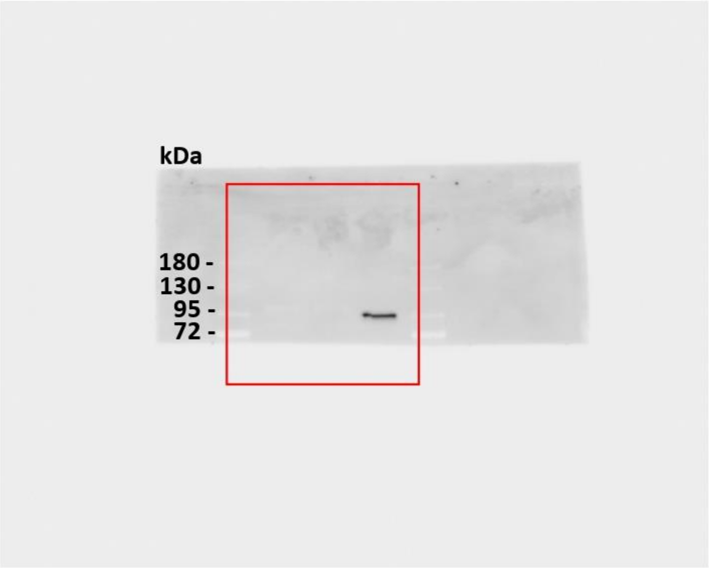

Figure 1B: Blot: Flag

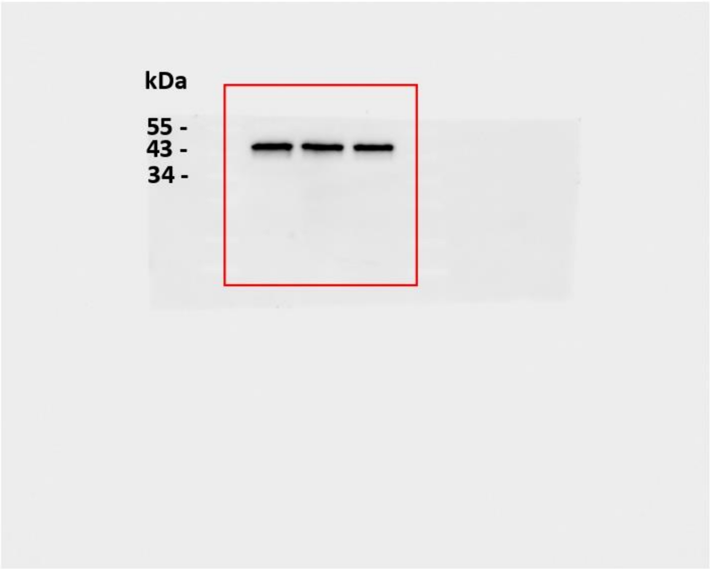

Figure 1B: Blot: β-Actin

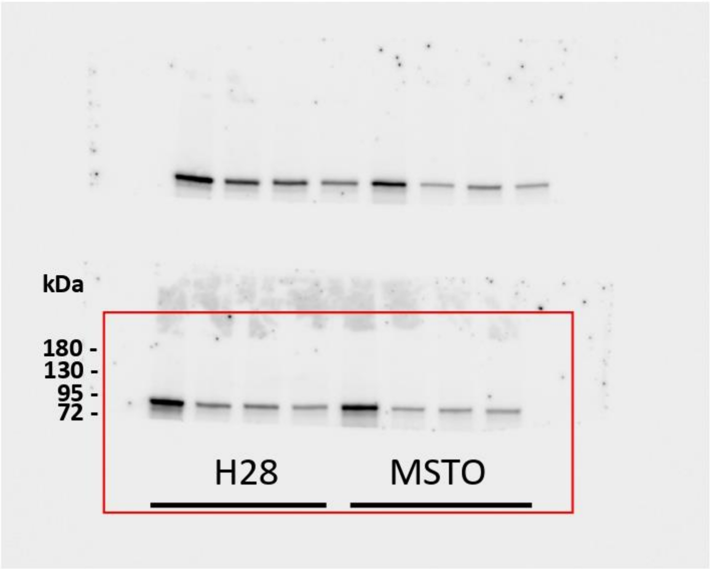

Figure 1C: Blot: STAT3

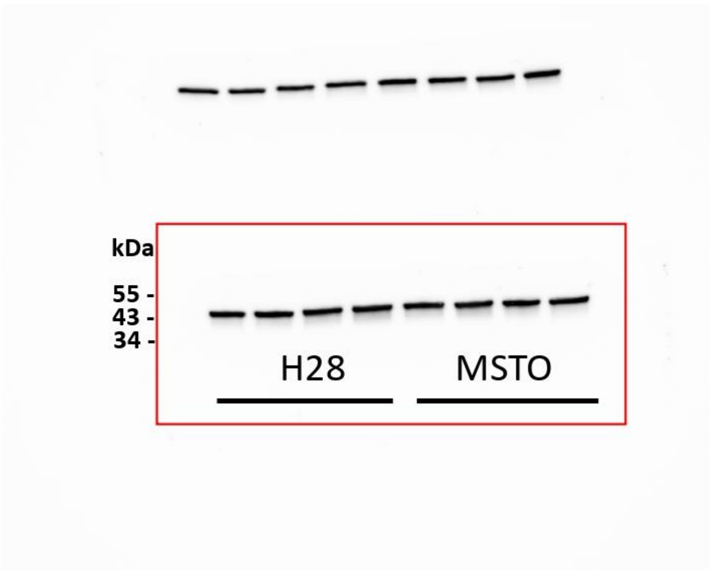

Figure 1C: Blot: β-Actin

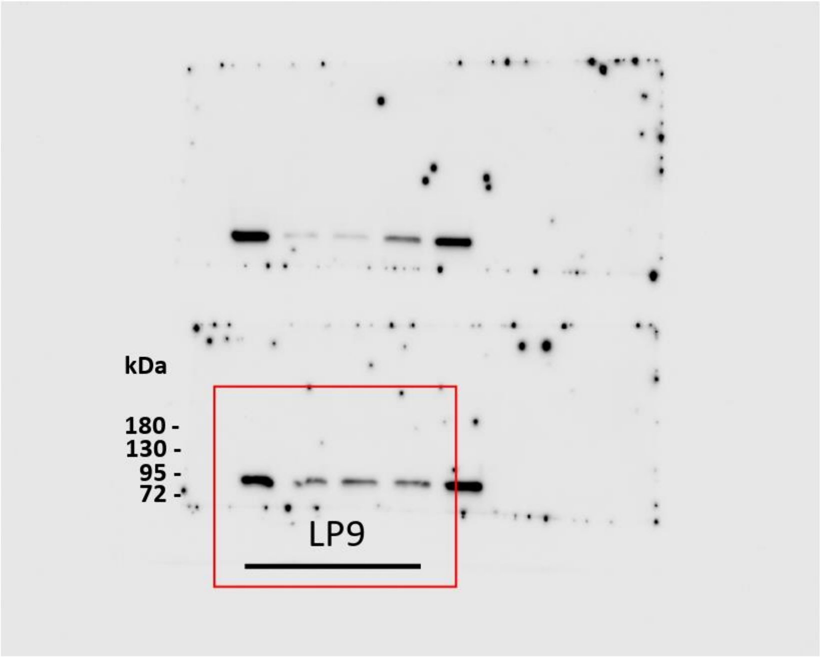

Figure 1C: Blot: STAT3

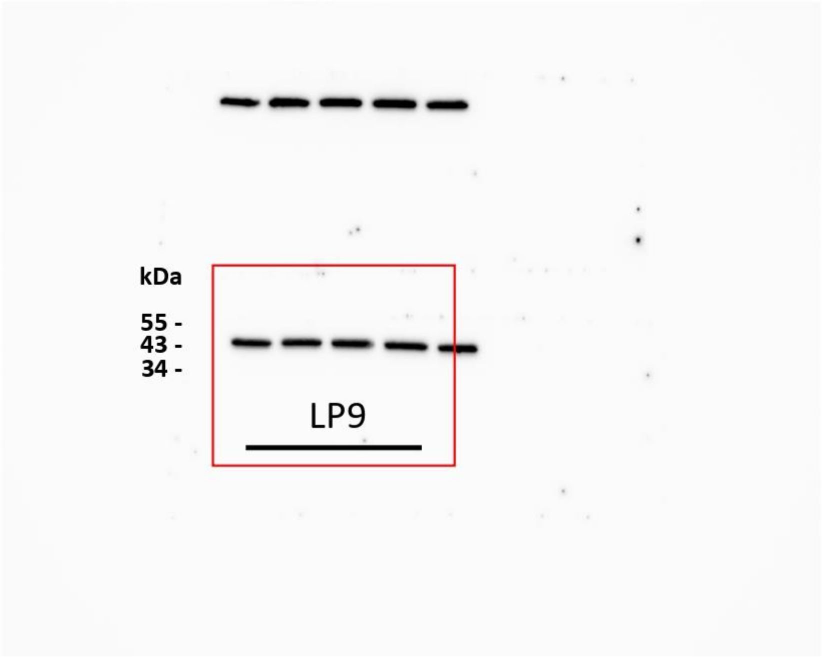

Figure 1C: Blot: β-Actin

Figure S1. Uncropped Western Blot images.

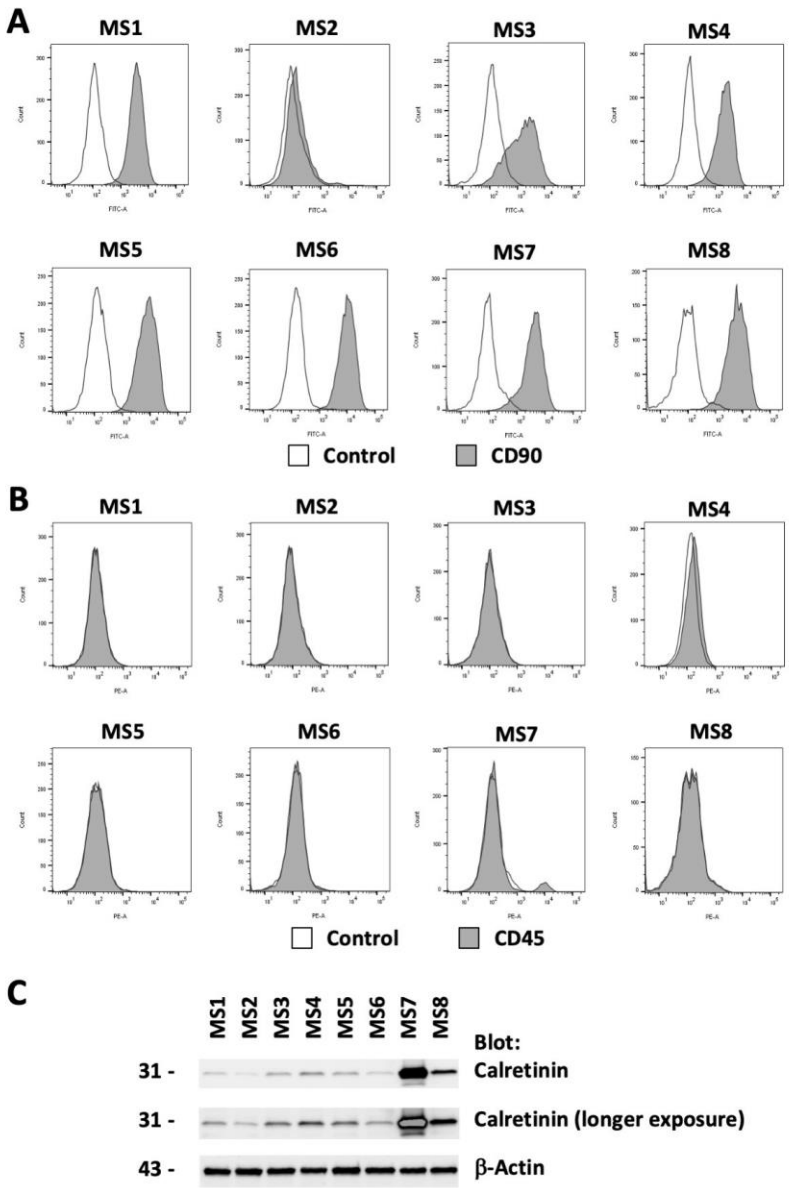

Figure S2. Characterization of MPM primary-derived cell lines. The cell surface expression of **A**, CD90 (eBio5E10, FITC-conjugated mouse anti-human CD90 (Thy-1), eBioscience/ThermoFisher) and **B**, CD45 (HI30, PE-conjugated mouse anti-human CD45, BD Pharmingen) was determined in cell lines by flow cytometry. **C**, protein expression of calretinin (SP13, ThermoFisher) was determined by immunoblotting and compared to expression of β-actin.

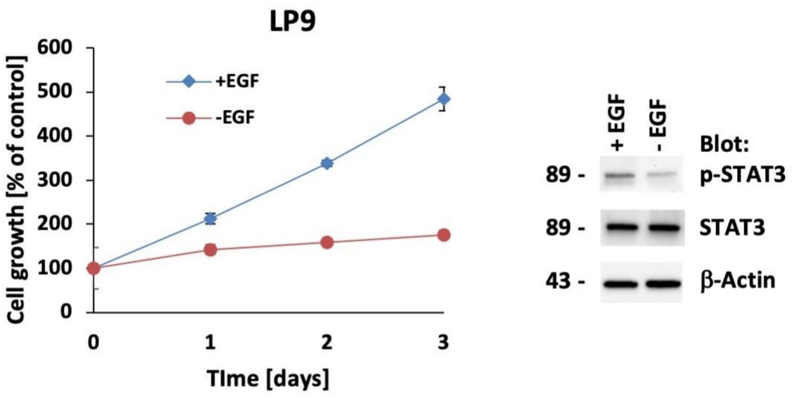

Figure S3. Cell growth and STAT3 phosphorylation in LP9 cells in response to EGF. Cell growth was measured for three days in the absence or presence of EGF, as indicated (left). Protein expression of STAT3, phospho-STAT3 (Tyr705) and β-actin was measured by immunoblotting (right).

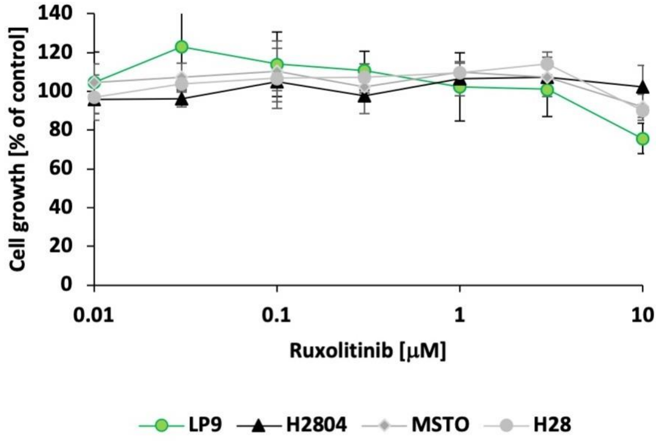

Figure S4. Cell growth of MPM cell lines in response to ruxolitinib. Cell growth was measured in a three-day assay (n=4) in response to ruxolitinib, as indicated.

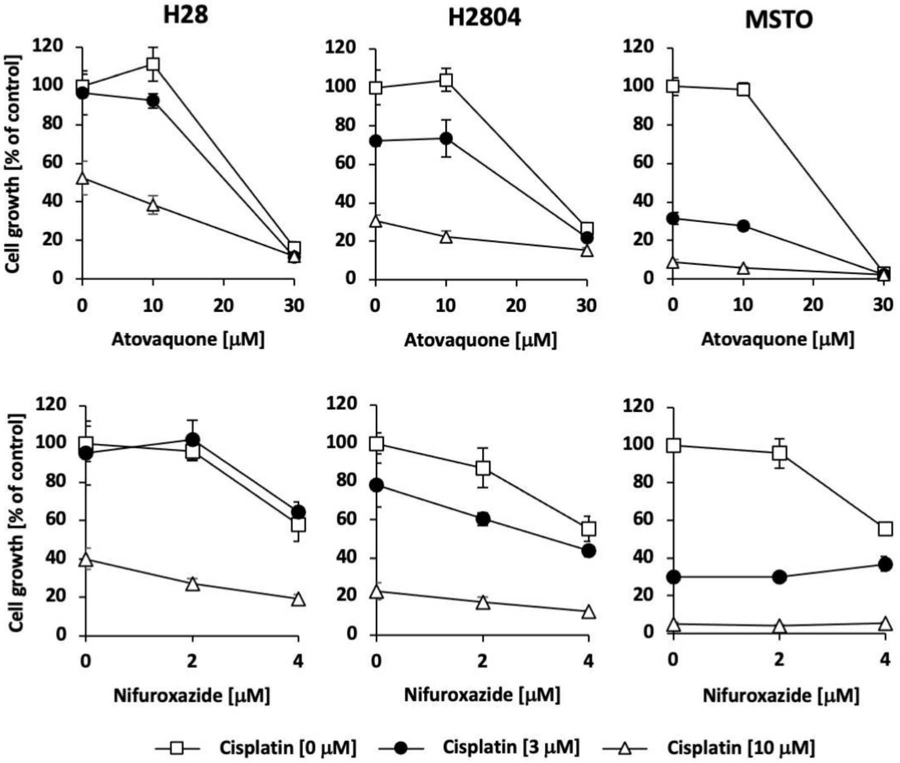

Figure S5. Combination of cisplatin with atovaquone or pyrimethamine in MPM cell lines. The MPM cell lines MSTO-211H (MSTO), H2052, H28, and H2804, as well as the non-transformed mesothelial cell line LP9, were treated with the indicated concentrations of atovaquone, nifuroxazide, or cisplatin for 72 hours, after which cell growth was measured (n=4).

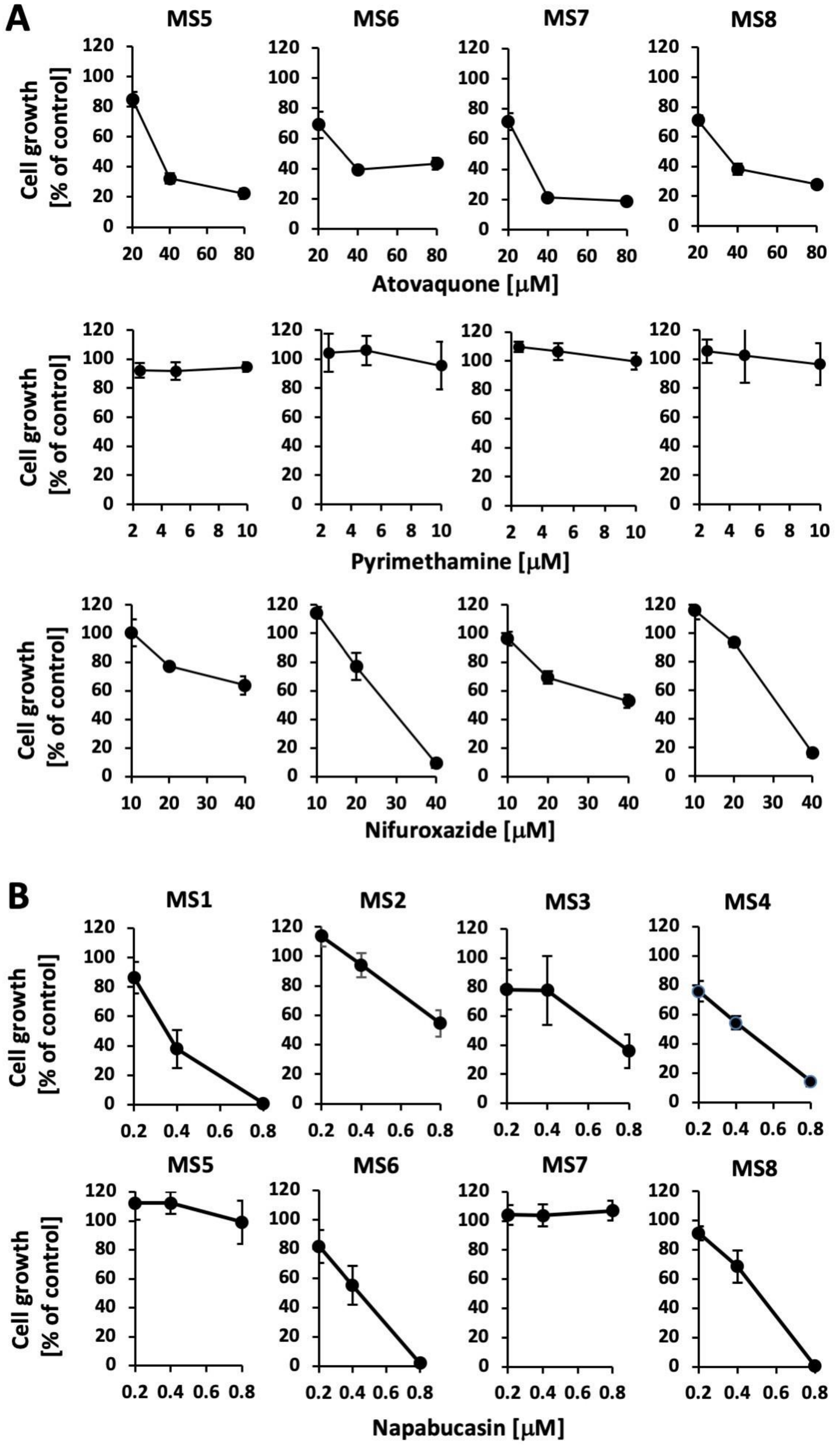

Figure S6. Cell growth of MPM primary-derived cells in response to atovaquone, pyrimethamine, nifuroxazide and napabucasin. A-B, cells were isolated from MPM specimen (MS1-MS8) and cell growth was measured in a three-day assay (n=4) in response to STAT3 pathway inhibitors, as indicated.

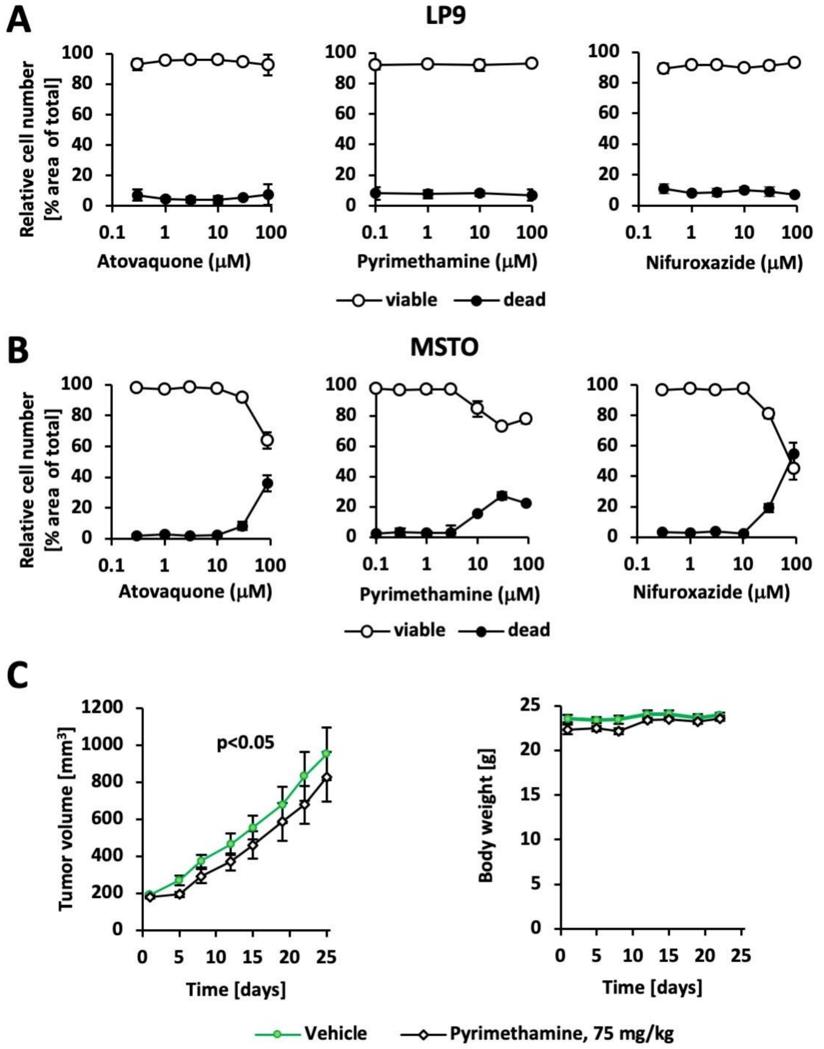

Figure S7. Targeting the STAT3 pathway in preclinical models of MPM. A, LP9 and **B**, MSTO-211H (MSTO) cells were treated for 4d with the indicated amounts of atovaquone, pyrimethamine, nifuroxazide or vehicle. Changes in the percent ratio of viable and dead cells were determined in a microfluidic 3D chamber (n=3). **C**, Mice implanted with MS4 tumor fragments were treated with 75mg/kg pyrimethamine or vehicle. Tumor volume and body weight were monitored.

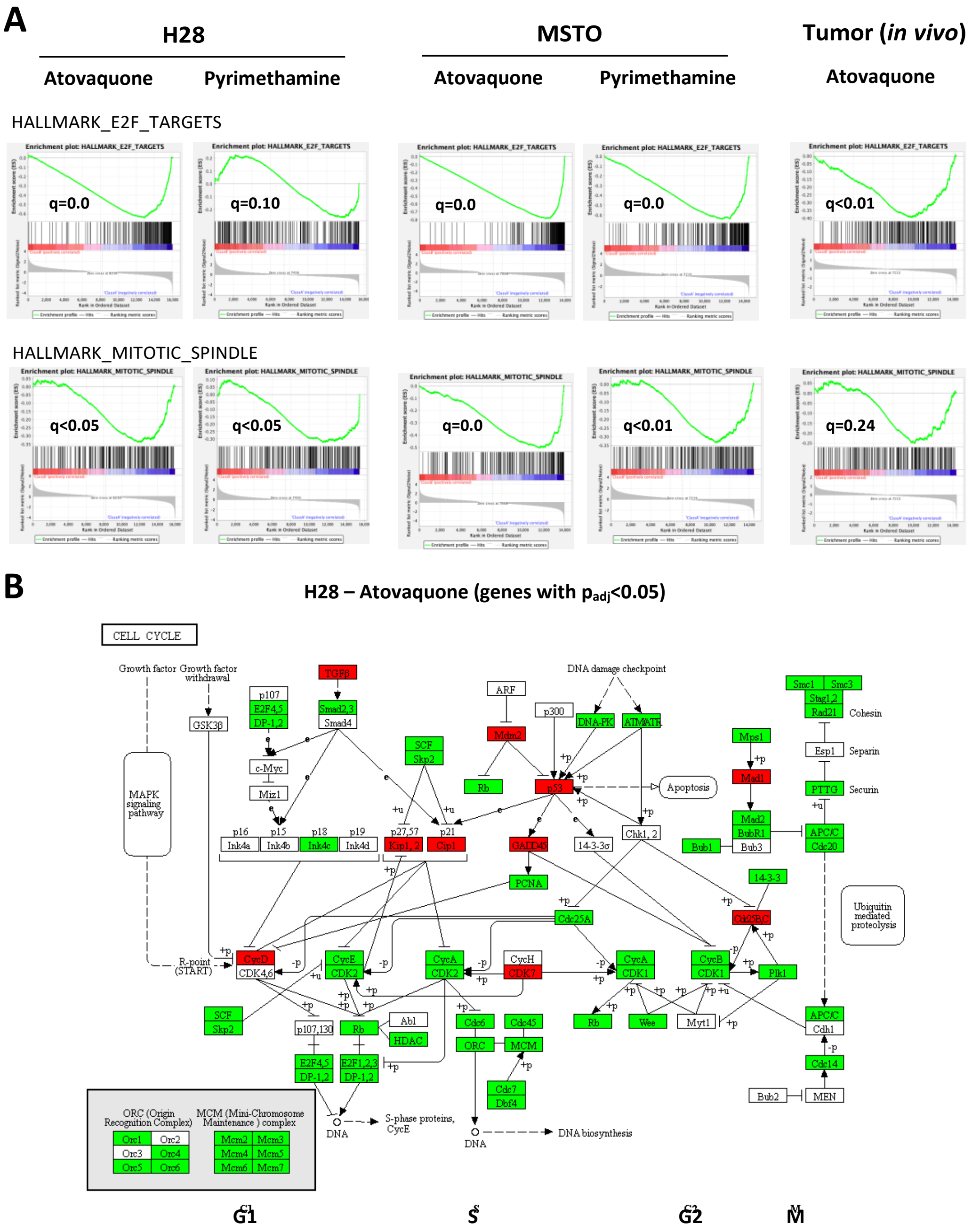

Figure S8. GSEA pathways downregulated by atovaquone and pyrimethamine. A, Enrichment plots of Hallmark pathways and FDR values for different models treated for 24h with either atovaquone (30 µM) or pyrimethamine (10 µM), as indicated. **B**, Significantly changed genes (fold change >2 or <0.5; p<0.05) in atovaquone-treated H28 cells were analyzed for their enrichment in the 'cell cycle' pathway using Pathview (upregulated genes - green, downregulated genes - red).

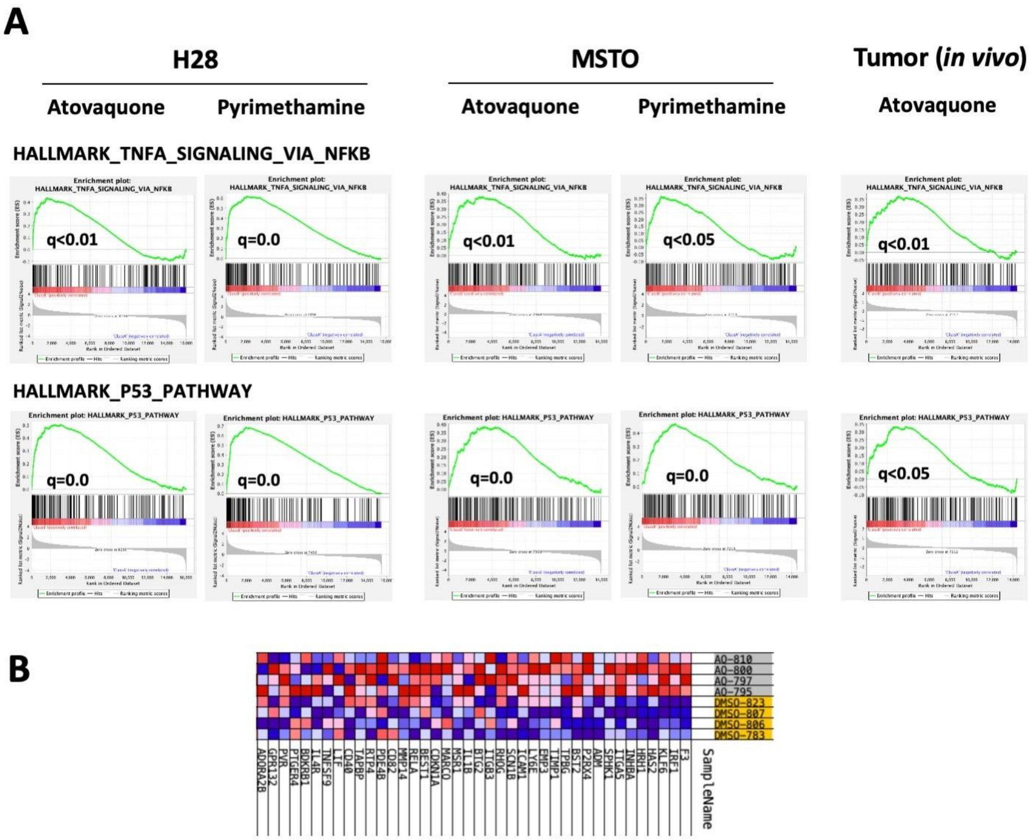

Figure S9. GSEA pathways upregulated by atovaquone and pyrimethamine. **A**, Enrichment plots of Hallmark pathways and FDR values for different models treated for 24h with either atovaquone (30 μM) or pyrimethamine (10 μM), as indicated. **B**, Enriched genes within the Hallmark Inflammatory Response pathway in M4 tumors treated with atovaquone or vehicle are shown (upregulated genes - blue, downregulated genes - red).

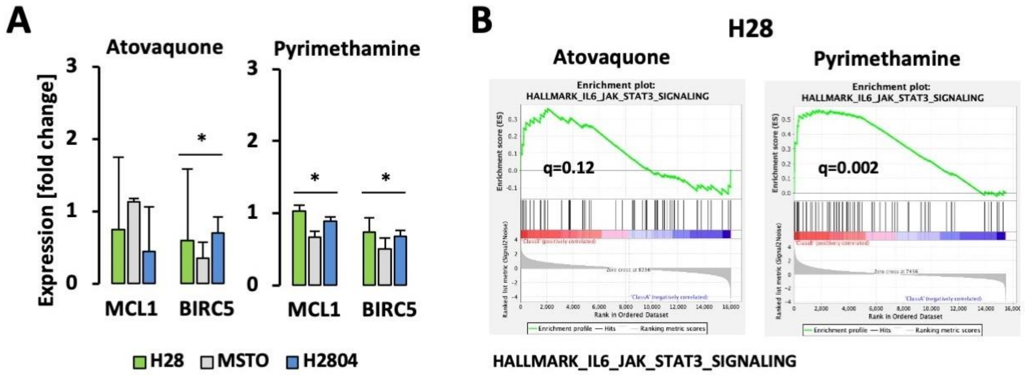

Figure S10. Atovaquone and pyrimethamine regulate the expression of STAT3 target genes. Cells were treated with either atovaquone (30 μM) or pyrimethamine (10 μM) for 24h. **A,** The expression of the STAT3 target genes MCL1 and BIRC5 was determined by RT-PCR in MPM cell lines (MSTO-211H (MSTO), H28, H2804) (n=2), as indicated (*significant differences with $p<0.05$). **B**, Enrichment plots of the HALLMARK_IL6_JAK_STAT3 pathway and FDR values for H28 cells, as indicated.

	Atovaquone	Pyrimethamine	Nifuroxazide
**LP9**	NR	NR	22.96 μM
**MSTO**	20.28 μM	1.22 μM	3.22 μM
**H2052**	25.96 μM	0.44 μM	1.72 μM
**H28**	21.88 μM	0.27 μM	2.9 μM
**H2804**	10.91 μM	2.96 μM	2.6 μM

Table S2. IC_{50} values for STAT3 pathway inhibitors. The IC_{50} values for atovaquone, pyrimethamine and nifuroxazide were determined in MPM cell lines (MSTO-211H (MSTO), H2052, H28, H2804) and LP9 control cells, as indicated. In some experiments the IC_{50} was not reached (NR) within the tested drug concentration of up to 30 μM.

UP	H28-Atov			H28-Pyr		
	NES	NOM p-val	FDR q-val	NES	NOM p-val	FDR q-val
HALLMARK_P53_PATHWAY	2.2	0.000	0.000	2.45	0.000	0.000
HALLMARK_TNFA_SIGNALING_VIA_NFKB	1.88	0.000	0.001	2.18	0.000	0.000
HALLMARK_INFLAMMATORY_RESPONSE	1.34	0.050	0.112	1.96	0.000	0.000
HALLMARK_COAGULATION	1.35	0.042	0.121	1.49	0.019	0.033
HALLMARK_MYOGENESIS	1.41	0.018	0.105	1.63	0.000	0.009
**DOWN**						
HALLMARK_G2M_CHECKPOINT	-2.95	0.000	0.000	2.48	0.000	0.000
HALLMARK_E2F_TARGETS	-3.03	0.000	0.000	-1.29	0.032	0.097
HALLMARK_MITOTIC_SPINDLE	-1.58	0.000	0.016	-1.62	0.000	0.000
HALLMARK_SPERMATOGENESIS	-1.48	0.010	0.037	-1.52	0.000	0.023
HALLMARK_ANDROGEN_RESPONSE	-1.78	0.000	0.005	-1.25	0.103	0.104
HALLMARK_PEROXISOME	-1.21	0.116	0.232	n/a	n/a	n/a

UP	MSTO-Atov			MSTO-Pyr		
	NES	NOM p-val	FDR q-val	NES	NOM p-val	FDR q-val
HALLMARK_P53_PATHWAY	1.84	0.000	0.006	2.11	0.000	0.000
HALLMARK_TNFA_SIGNALING_VIA_NFKB	1.85	0.000	0.009	1.63	0.000	0.031
HALLMARK_INFLAMMATORY_RESPONSE	1.13	0.209	0.336	1.26	0.076	0.244
HALLMARK_COAGULATION	1.1	0.281	0.351	1.35	0.068	0.177
HALLMARK_MYOGENESIS	1.47	0.012	0.064	0.86	0.793	0.855
**DOWN**						
HALLMARK_G2M_CHECKPOINT	-3.74	0.000	0.000	-3.17	0.000	0.000
HALLMARK_E2F_TARGETS	-3.88	0.000	0.000	-3.22	0.000	0.000
HALLMARK_MITOTIC_SPINDLE	-2.48	0.000	0.000	-1.68	0.000	0.007
HALLMARK_SPERMATOGENESIS	-2.13	0.000	0.000	-1.66	0.003	0.007
HALLMARK_ANDROGEN_RESPONSE	-1.26	0.100	0.145	-1.52	0.009	0.018
HALLMARK_PEROXISOME	-1.49	0.006	0.024	-1.76	0.000	0.003

UP	MS4-Tumor		
	NES	NOM p-val	FDR q-val
HALLMARK_P53_PATHWAY	1.57	0.000	0.031
HALLMARK_TNFA_SIGNALING_VIA_NFKB	1.74	0.002	0.007
HALLMARK_INFLAMMATORY_RESPONSE	1.56	0.005	0.027
HALLMARK_COAGULATION	1.72	0.000	0.009
HALLMARK_MYOGENESIS	1.5	0.005	0.044
**DOWN**			
HALLMARK_G2M_CHECKPOINT	-1.92	0.000	0.002
HALLMARK_E2F_TARGETS	-1.95	0.000	0.004
HALLMARK_MITOTIC_SPINDLE	-1.25	0.062	0.243
HALLMARK_SPERMATOGENESIS	-1.58	0.009	0.048
HALLMARK_ANDROGEN_RESPONSE	-1.48	0.010	0.080
HALLMARK_PEROXISOME	-1.33	0.063	0.151

FDR q-val<0.250

Table S8. Common up- and down-regulated GSEA pathways. Common GSEA pathways (FDR q-value <0.250, green) were identified in MSTO-211H (MSTO) and H28 cells treated for 24h with pyrimethamine (10 μM) or atovaquone (30 μM) as well as in atovaquone treated MS4 tumors.
